# Cooper’s Ferry/Nipéhe, stratigraphic integrity, and confirming early inland dispersal: Evaluating peopling of the Americas modeling

**DOI:** 10.1126/sciadv.aek3138

**Published:** 2026-11-04

**Authors:** Loren G. Davis, David B. Madsen

**Affiliations:** ^1^ Department of Anthropology, Oregon State University, 203 Waldo Hall, Corvallis, OR 97331, USA.; ^2^ Department of Anthropology, University of Nevada-Reno, 512 Ansari, Reno, NV 89557, USA.

## Abstract

Statistical models shape debates over the timing and geography of the initial peopling of the Americas, but their value depends on testing their assumptions against archaeological evidence. We examine the Apparent Stratigraphic Integrity Index (ASI), used to infer postdepositional mixing at Late Pleistocene sites and support a relatively late occupation of temperate North America. ASI describes vertical discontinuity in binned artifact-frequency profiles; it does not directly measure artifact movement or stratigraphic integrity. At Cooper’s Ferry/Nipéhe, Idaho, the 5-centimeter absolute-elevation bins used to calculate ASI do not reflect the actual stratigraphic positions of artifacts across the irregular LU2 surface on which LU3 loess accumulated. The resulting ASI value cannot support claims of large-scale vertical mixing. Independent chronological, stratigraphic, pedogenic, archaeological, and excavation evidence supports the integrity of the ∼16,000–calibrated-years-before-the-present occupation. Cooper’s Ferry/Nipéhe provides early inland evidence expected under coastal-migration models, showing that models should be evaluated against archaeological evidence rather than used to override it.

## INTRODUCTION

For more than 50 years, researchers have used computer simulations, statistical models, and mathematical equations to investigate questions about the initial peopling of the Americas, including the timing and location of entry, rates of population growth, and the pace of geographic expansion. During the latter half of the 20th century, many of these models examined how small populations associated with the Clovis Paleoindian Tradition (CPT) could have spread rapidly across the Americas after ∼14,000 calibrated years before the present (cal yr B.P.) (*1*–*11*). As evidence for sites predating the CPT accumulated during the first two decades of the 21st century, some models instead assessed whether those earlier occupations were consistent with proposed dispersal histories and, in some cases, were used to question their reported ages (*12*–*14*). Yet researchers have rarely examined the assumptions built into these models or their suitability for evaluating particular archaeological sites.

Here, we use Cooper’s Ferry/Nipéhe as an archaeological test case for evaluating three statistical and demographic models that address different dimensions of the initial peopling of the Americas. We begin with the Apparent Stratigraphic Integrity Index (ASI) developed by Surovell *et al.* (*14*) and used to question the stratigraphic integrity of several Late Pleistocene sites and to support a relatively late initial occupation of mid-latitude North America. We examine what the ASI actually measures, the assumptions required to compare its values among different sites, and whether its application to Cooper’s Ferry/Nipéhe adequately represents the site’s stratigraphic record. We then consider the implications of the resulting archaeological evidence for two earlier models developed by Surovell. Surovell’s (*12*) coastal-migration model predicted that inland dispersal should occur in western North America before coastal populations reached southern South America and specifically identified the Columbia River as an accessible corridor into the continental interior. Surovell’s (*15*) demographic model, although originally developed to explain the rapid expansion of Clovis populations, examined the rates of mobility and population growth necessary for human populations to spread rapidly across North America. Together, these models allow us to consider a broader methodological question: How should formal models of initial human dispersal be evaluated when archaeological evidence becomes available that can directly test their assumptions and predictions?

## RESULTS AND DISCUSSION

### The ASI

Surovell *et al.* (*14*) develop the ASI statistic, which, they contend, captures the variability in the degree of mixing in multicomponent archaeological sites as reflected by changes in vertical artifact distributions. They then apply the ASI to vertical artifact distributions from five pre-13,000–cal yr B.P. sites in Alaska and compare them with distributions from three sites south of the ice sheets that date to more than 15,000 cal yr B.P. Based on differences in ASI among these sites, they conclude that their “…findings support the hypothesis that the first human arrival to the New World occurred by at least 14,200 BP in Beringia and by approximately 13,000 BP in the temperate latitudes of North America” [(*14*), p. 12].

#### What the ASI actually measures

The ASI is not a direct measure of artifact movement, postdepositional disturbance, or stratigraphic integrity. It is a numerical description of the shape of a vertical artifact-frequency profile. To calculate it, artifact counts are placed in successive excavation levels or elevation bins, consecutive sterile levels are arbitrarily collapsed into a single zero value, and the absolute difference in artifact count between each pair of adjacent bins is summed and standardized by the mean artifact count for the sequence. Put simply, the ASI arbitrarily measures how sharply artifact frequencies rise and fall from one vertical bin to the next.

High ASI values indicate abrupt changes in artifact frequency between adjacent bins, which are assumedly (but not necessarily) adjacent stratigraphic contexts, such as dense concentrations vertically separated by low-density or sterile intervals. Low ASI values indicate more gradual changes from bin to bin, producing a smoother and more continuous vertical distribution. The ASI therefore only measures the degree of vertical discontinuity in artifact counts; it does not identify the cultural or natural site-formation processes that produced that pattern.

Surovell *et al.* (*14*) link ASI values to general processes of stratigraphic mixing through simulations that begin by modeling the placement of artifacts from repeated occupations on discrete depositional surfaces and then imposing specified rates of vertical artifact dispersal. Vertical movement is therefore a fundamental assumption of the model, not an independent finding derived from geoarchaeological observations of assuredly distinctive site-formation processes at the archaeological sites to which the index is applied. The simulations move artifacts associated with a discrete occupation surface upward and downward, spreading a relatively narrow concentration into a broader vertical band. As artifacts from surfaces above and below are similarly dispersed, the bands overlap and merge into a more homogeneous vertical cloud of artifacts. Artifact frequencies then change less sharply between adjacent bins, producing lower ASI values.

The ASI simulations show only how the index behaves under a set of conditions defined by the model. No study has tested the simulations against an actual archaeological site in a way that accounts for its specific geological context, stratigraphic characteristics, and formation processes, and no study has shown that a low ASI value reliably reflects vertical artifact movement or accurately measures the magnitude of that movement. Applying the ASI to a real site therefore requires an additional (but unexplained) interpretive step: The analyst must assume that a continuous artifact-frequency profile results from some kind of postdepositional mixing rather than from some other combination of depositional, behavioral, and recovery processes. Similar profiles could result from closely repeated occupations during relatively continuous sediment accumulation, differences in occupation duration or discard intensity, shifting activity areas, changing sedimentation rates, excavation-bin construction, sample size, or recovery conditions. Conversely, a disturbed deposit could theoretically retain sharp changes in artifact frequency, which the ASI would treat as a hallmark of high stratigraphic integrity.

The term apparent is therefore central to the index. The ASI can identify a pattern in vertically ordered artifact counts and may generate a hypothesis about possible mixing, but it cannot by itself demonstrate the magnitude, direction, mechanism, or archaeological consequences of artifact movement. Those questions require independent evidence from stratigraphy, sedimentology, feature preservation, chronometry, pedogenesis, and the spatial patterning of excavated objects.

Surovell *et al.* (*14*) explicitly acknowledge several limitations on the use of the ASI. They state, “We do not mean to imply that this function can be used to describe all archaeological cases…” [(*14*), p. 2]. They also note that “One disadvantage of the ASI…is that its value can vary depending upon an arbitrary choice of thickness of excavation levels or elevation bins” [(*14*), p. 5]. In addition, they caution that “it is difficult to determine the stratigraphic integrity of any archaeological site with a small assemblage,” particularly those with “less than a mean of approximately 30 artifacts per level” [(*14*), p. 7]. Last, they explain that “the use of the term ‘apparent’…[in the ASI name]…is because frequently reoccupied but minimally disturbed sites could theoretically exhibit similar stratigraphic artifact distributions to highly disturbed sites” [(*14*), p. 3]. Despite these caveats, Surovell *et al.* [(*14*), p. 3] contend that the ASI provides “…a reasonable approximation of the relative degree to which artifacts at different depths are subject to post-depositional disturbance processes.”

Whether that approximation is reasonable depends on the conditions under which ASI values are compared. In addition to the limitations of the model described by Surovell *et al.* (*14*), there are several other conditions that must be assumed, held constant, or independently demonstrated before differences in ASI values can be interpreted as differences in the degree of mixing:

1) Vertical artifact movement must be the principal cause of smoothing in the artifact-frequency profiles being compared. In the ASI simulations, lower values result from imposed upward and downward artifact displacement. At an actual archaeological site, however, a smoother profile could also result from repeated occupations during relatively continuous sediment accumulation, differences in occupation duration or discard intensity, changing sedimentation rates, excavation-bin construction, sample size, or recovery conditions.

The index also cannot produce a perfect score for a real archaeological assemblage. Artifact elevations are first grouped into arbitrary 5-cm bins, which necessarily combine objects deposited at slightly different vertical positions within each bin. As a result, every site will have an ASI value below 1.0 and, in this limited ASI-defined sense, will lack complete stratigraphic integrity to a greater or lesser degree. The computed ASI values reported by Surovell *et al.* (*14*) range from 0.045 to 0.676, indicating apparent vertical smoothing even at the sites they regard as most intact. This is particularly revealing in the case of Broken Mammoth, one of the early Beringian sites that Surovell *et al.* [(*14*), p. 1] collectively describe as showing “excellent stratigraphic integrity,” which has an ASI value of only 0.367. Their simulation results, however, indicate that relatively intact distributions with little mixing generally produce ASI values between 0.4 and 1.0, whereas highly mixed sites tend to fall below 0.3. Broken Mammoth therefore falls below 0.4 despite being treated elsewhere in their analysis as a minimally disturbed and stratigraphically straightforward site. This inconsistency illustrates that ASI values do not map cleanly onto the qualitative categories of stratigraphic integrity used elsewhere in their analysis. More fundamentally, the index provides a relative measure of discontinuity among already binned artifact-frequency profiles; it does not establish whether a particular value reflects postdepositional movement or measure how much movement occurred.

2) The sites being compared must have broadly comparable depositional, pedogenic, and chronostratigraphic histories. Artifact movement in gradually aggrading loess need not resemble movement in a clay vertisol, where desiccation cracks can vertically displace artifacts. In loess, gradual sediment accumulation may bury and preserve artifacts at or near their original depositional positions. In contrast, shrink-swell processes in vertisols can create cracks into which artifacts may fall and subsequently be displaced.

The ASI also reduces differences in the thickness and duration of sterile intervals between occupations. Consecutive sterile levels are collapsed into a single zero value before the index is calculated. Thus, an archaeological sequence in which occupations are separated by 1.0 m of sterile sediment, or 20 5-cm bins, may produce an ASI value similar to one in which occupations are separated by only a single sterile 5-cm interval. Yet the same degree of vertical artifact movement would have very different implications in these two cases. Modest movement through a meter-thick sterile deposit could leave occupations clearly separated and archaeologically distinguishable, whereas similar movement across a single 5-cm sterile interval could substantially intermingle artifacts from successive occupations. As Surovell *et al.* (*14*) recognize, the ASI does not account for this difference.

3) The artifact assemblages being compared must respond similarly to postdepositional processes. The ASI does not distinguish large objects, such as Clovis or Haskett projectile points, from small objects such as pressure flakes. In the clay-vertisol example, small artifacts may be more readily displaced into desiccation cracks than larger objects. A combined artifact profile may therefore yield a low ASI value although larger diagnostic artifacts retain a distinct and stratigraphically meaningful distribution.

This appears particularly relevant at Gault, which does contain shrink-swell clays and is one of the Late Pleistocene sites south of the continental ice sheets that Surovell *et al.* (*14*) contend lacks stratigraphic integrity. There, small objects have a different vertical profile than larger artifacts, a pattern that Surovell *et al.* [(*14*), p. 12] suggest may “…provide[s] independent evidence for stratigraphic integrity.” The reported 0.219 “intermediate” ASI score may therefore reflect, at least in part, the inclusion of artifact classes with different susceptibilities to movement. Whether an analysis limited to larger diagnostic artifacts would produce a substantially different value or raise the ASI above 0.40, within the range that Surovell *et al.* (*14*) associate in their simulations with relatively intact distributions, remains to be determined. Although this large/small distinction is especially apparent in vertisols, differences in artifact size, mass, and shape are likely relevant under most sedimentary conditions.

4)Excavation blocks must sample successive occupations in broadly comparable ways. Occupations need not have the same duration, discard intensity, or spatial organization from one interval to the next. A short visit may leave few objects, whereas a longer stay may produce a dense artifact concentration, even where both occupations are intact. Similarly, the principal location of activity may shift across a site through time.

Unless a multicomponent site is excavated across its full spatial extent, a rare circumstance, a fixed excavation block may sample the center of one occupation, the edge of another, and miss a third altogether. These differences can produce strong variation in artifact frequencies through time without any postdepositional mixing. As illustrated in Fig. 1, shifts in the horizontal placement of otherwise intact occupation areas can produce alternating, smoothly declining, or intermittent artifact-frequency profiles within a fixed excavation block. Such profiles may therefore reflect differences in the spatial relationship between successive occupations and the excavated sample rather than differences in stratigraphic integrity.

**Fig. 1.**
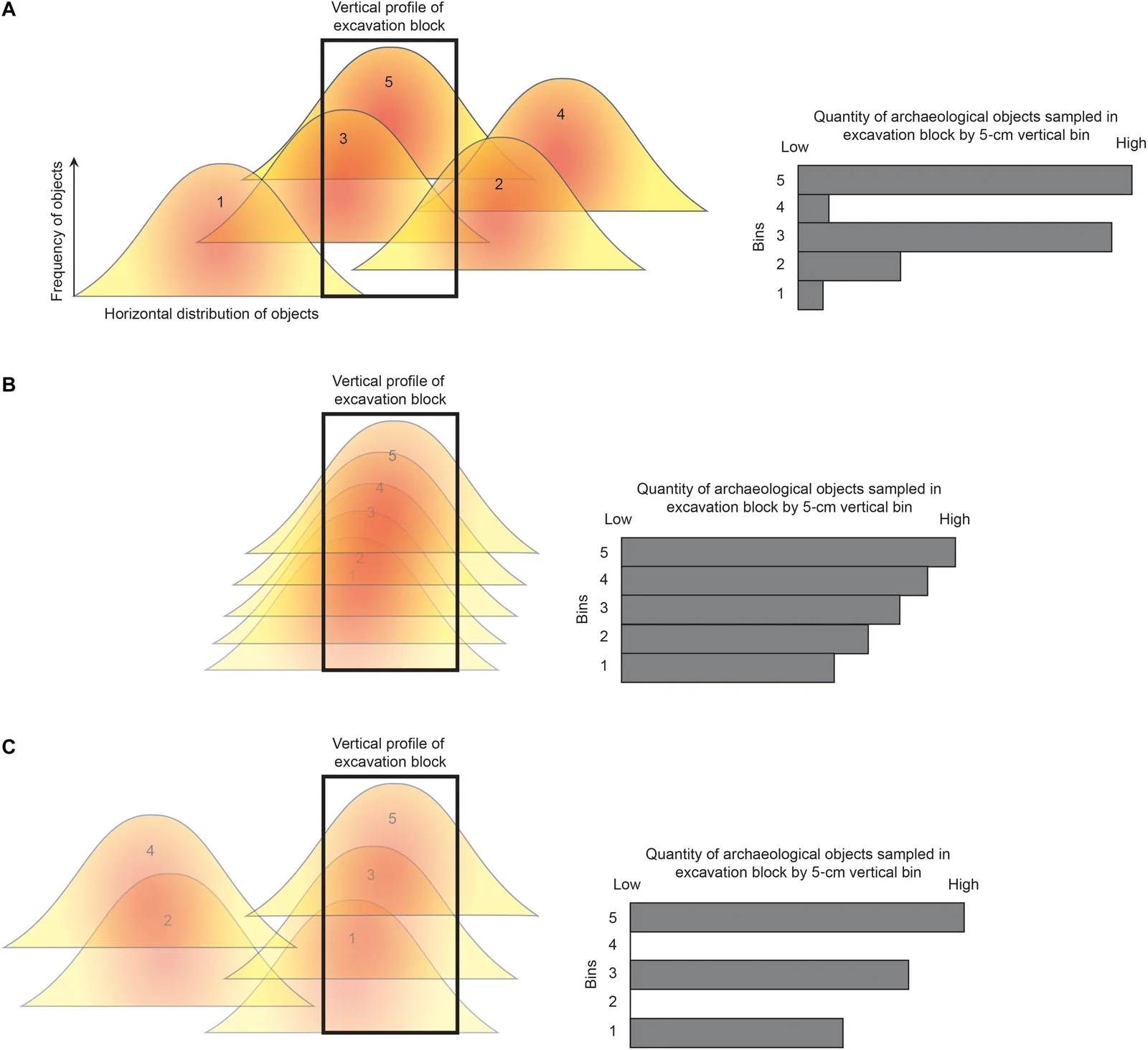
Spatial sampling effects on vertical artifact frequencies. Hypothetical examples illustrating how variation in the horizontal placement of sequential occupation areas can affect the number of archaeological objects recovered within a fixed excavation block. The height of each bell curve represents the frequency of objects deposited within an occupation area, whereas its width represents the horizontal extent of the associated artifact scatter. Numbered curves represent successive occupations, and the rectangle indicates the vertical profile sampled by the excavation block. The base of each bell curve corresponds to the stratigraphic position of that occupation assemblage within the excavation block. Bar graphs at right show the resulting number of archaeological objects recovered in each 5-cm vertical bin. (**A**) Sequential occupations distributed irregularly across the site, but generally near the excavation area, produce alternating high and low artifact frequencies in the excavated sample. (**B**) Repeated occupations centered in one area but gradually shifting away from the excavation block produce a smooth decline in artifact frequencies. Conversely, occupations gradually shifting toward and into the excavation block would produce a smooth increase. (**C**) Repeated occupations distributed across different parts of the site produce an intermittent pattern of artifact presence and absence within the excavation block. In this example, the site was occupied repeatedly, but the selected excavation area samples only part of the spatial distribution of human activity through time.

These conditions do not mean that the ASI lacks descriptive value. It can summarize the degree of vertical discontinuity in an artifact-frequency profile and may help generate hypotheses about possible mixing. But it is a limited solitary measure for comparing stratigraphic integrity among sites with different depositional histories, occupational histories, artifact assemblages, excavation strategies, and site-formation processes. We could go on, but the central point is that the ASI is a crude and potentially unreliable stand-alone measure for comparing relative stratigraphic integrity among sites with different depositional histories, occupational histories, artifact assemblages, and site-formation processes.

### Stratigraphic integrity at the Cooper’s Ferry/Nipéhe site

Although Surovell *et al.* (*14*) developed the ASI as a comparative statistic, its validity depends first on whether it has been appropriately applied to the site-specific datasets being compared. We therefore examine their application of the ASI to Cooper’s Ferry/Nipéhe, one of the three Late Pleistocene sites south of the continental ice sheets included in their analysis.

#### Site setting and chronological record

The Cooper’s Ferry/Nipéhe site (10IH73) lies on an alluvial terrace at the confluence of Rock Creek and the lower Salmon River in western Idaho, within the traditional homeland of the Nimiipuu (Nez Perce). Excavations conducted in 1997 and from 2009 to 2018 documented a stratified Late Pleistocene–early Holocene archaeological sequence in two large block excavations, Areas A and B (*16*–*18*).

The earliest cultural materials occur within Hammer Creek Loess associated with the Rock Creek Soil. Bayesian modeling places the onset of occupation in Area A at 16,560 to 15,280 cal yr B.P., whereas radiocarbon-dated pit features in Area B date between 16,675–15,240 and 15,775–15,620 cal yr B.P. (*17*, *18*). These deposits contain cultural pit features, a hearth, faunal remains, lithic debitage, flake tools, cores, blades, bifaces, and bifacially flaked stemmed projectile points, including 14 points from the ∼15,785 cal yr B.P. Area B assemblage (*17*–*20*). American Upper Paleolithic (AUP) assemblages represent the initial occupation at the site dating from ∼16,000 to 13,400 cal yr B.P. (*21*), followed by Western Stemmed Tradition occupations that continue through the early Holocene to ∼9500 cal yr B.P. (*16*, *17*, *19*). Together, the site’s lithostratigraphic, pedostratigraphic, archaeological, radiocarbon, and Bayesian chronological evidence provides a high-resolution record of Late Pleistocene occupation south of the continental ice sheets and early stemmed-point technology in western North America.

#### The Cooper’s ferry/Nipéhe ASI dataset and elevation-binning problems

Surovell *et al.* (*14*) considered only three sites south of the continental ice sheets because “appropriate data were not available” for other sites older than ∼14,000 cal yr B.P. Cooper’s Ferry/Nipéhe should likewise not have been treated as an appropriate dataset for this purpose. At the time Surovell *et al.* (*14*) assembled their data, analysis of the excavated materials was ongoing, and key information necessary to evaluate the stratigraphic relationships of the artifacts was not represented in the published dataset they used.

Surovell *et al.* [(*14*), supplementary information, p. 5] drew the vertical artifact distribution from table S4 of Davis *et al.* (*17*). They stated that they included “all piece-plotted chipped stone artifacts and bone outside of cultural features” from LU3. Because they observed “no discrete cultural levels and no stratigraphic tilt” in the backplot and believed that published profiles showed “little if any slope,” they did not correct for sloping beds and binned the artifacts and bone into 18 artificial 5-cm elevation intervals ranging from 411.00–411.05 to 411.85–411.90 masl, creating an invented stratigraphy not recognized in the actual excavations.

The problem is not simply whether the deposits slope. Rather, the analysis treats absolute artifact elevations across a broad excavation block as though they represent equivalent stratigraphic positions. LU3 consists of relatively continuously deposited, massive loess with no apparent internal stratification. These aeolian sediments accumulated over the irregular upper surface of culturally sterile LU2 alluvial sand. Within the mapped profile shown by Davis *et al*. [(*17*), p. 893] (Fig. 2A), the profile used by Surovell *et al.* (*14*), the LU2 surface varies approximately between 411.150 and 411.450 masl, or a vertical difference of 30 cm (Fig. 2A). The maximum difference between the highest and lowest recorded elevations of the LU2/LU3 contact surface in the larger Area A excavation block is 40.3 cm (Fig. 2B). The upper boundary of LU3 is an erosional unconformity.

**Fig. 2.**
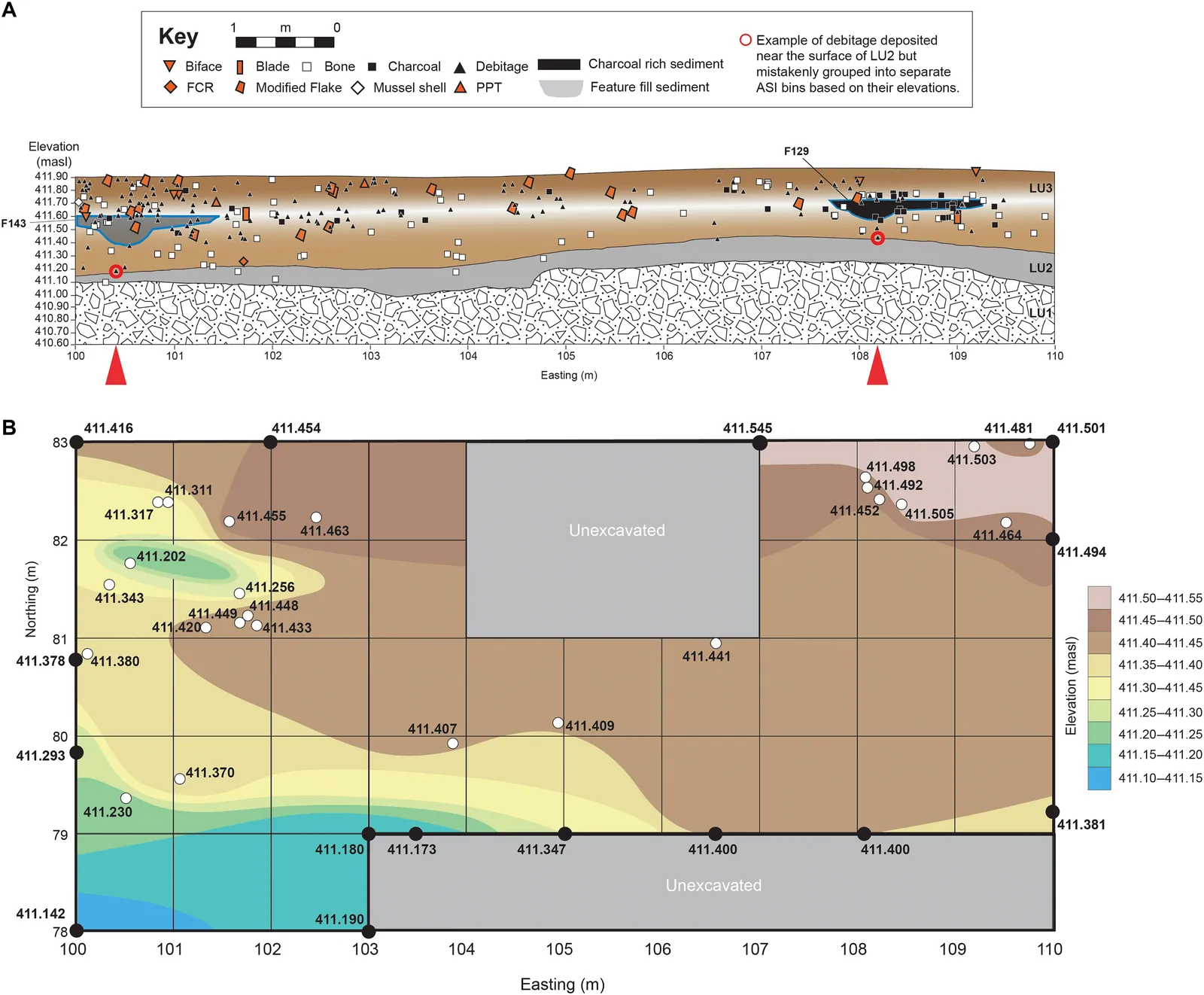
Lowermost LU3 artifacts and LU2 surface topography. (**A**) Stratigraphic profile of LU3 in Area A at Cooper’s Ferry/Nipéhe, showing in situ items mapped outside cultural pit features. Red arrowheads identify two circled pieces of debitage discarded immediately above the buried LU2 surface that were probably contemporaneous. (**B**) Contour plan map of the LU2 surface, conformably overlain by LU3 loess, showing elevations ranging from 411.142 to 411.545 masl. Mapped artifact locations (white circles) indicate the elevations of 24 items identified within the lowest 5 cm of LU3 directly above LU2.

The Rock Creek Soil is the only broadly recognizable pedostratigraphic marker within LU3. It developed within the loess after deposition and includes a diffuse Btk horizon whose limits cannot be mapped as sharp, laterally continuous contacts. The diffuse horizontal white line in Fig. 2A (*17*) marks only the approximate position of that Btk horizon. As a pedogenic subsoil horizon, it is neither a depositional surface nor a stratigraphic contact in the loess, and it provides no measure of the configuration of the internal paleosurfaces on which artifacts were discarded. It therefore cannot support an inference that the relevant depositional surfaces were horizontal or that paleosurface relief across Area A was negligible.

Table S4 of Davis *et al.* (*17*) reports the horizontal and vertical positions of LU3 artifacts mapped to the nearest millimeter across the 10 m–by–5 m Area A excavation block. It does not establish which mapped artifacts were deposited on shared paleosurfaces or how individual artifact elevations relate to the irregular underlying LU2 surface. As loess accumulated over that surface, successive depositional surfaces would have conformed, at least, in part, to its inherited underlying topography. Thus, two artifacts deposited on the same loess surface in different parts of Area A could differ in absolute elevation by many centimeters (Fig. 2).

Binning artifacts solely by absolute elevation can combine artifacts deposited on different paleosurfaces while separating artifacts deposited on the same paleosurface. In this respect, the 5-cm horizontal bins used by Surovell *et al.* (*14*) create artificial vertical groupings rather than representing actual stratigraphic intervals across the excavation block. This is not simply a matter of reduced precision. Because the aggrading LU3 paleosurface varied in elevation across the excavation area and was not the planar surface assumed by Surovell *et al.* (*14*), artifacts discarded contemporaneously on that surface do not necessarily occur at the same absolute elevation, whereas artifacts at the same absolute elevation may derive from different depositional surfaces and occupation intervals. The horizontal binning procedure therefore reverses the relationships that must be evaluated to assess stratigraphic integrity: It artificially spreads contemporaneous artifacts across multiple ASI bins while potentially combining artifacts that were not deposited together (Table 1). Because Surovell *et al.* (*14*) did not account for the topography of the LU3 paleosurface or the local stratigraphic position of individual artifacts, their ASI bins are not a valid representation of artifact distributions through the deposit. Any ASI value derived from those bins consequently measures a distribution created by the binning procedure rather than the actual stratigraphic distribution of artifacts at Cooper’s Ferry/Nipéhe. For example, the 24 items mapped in situ in the 5 cm immediately above the LU3-LU2 stratigraphic contact (Fig. 2B) were incorrectly assigned by Surovell *et al.* [(*14*), table S3] to seven separate 5-cm ASI bins ranging from bin 5 (411.20 to 411.25) to bin 11 (411.50 to 411.55). In short, the “stratigraphic” elevational bins of Surovell *et al.* (*14*) are artificial inventions, as is any resulting ASI score for Cooper’s Ferry/Nipéhe.

**Table 1.** Mapped basal LU3 items and assigned ASI bins. Items mapped in LU3 within the lowest 5 cm above the LU2/LU3 contact are compared with the 5-cm ASI elevation bins assigned by Surovell *et al.* (*14*). The 24 items mapped just above the surface of LU2, shown in bold, were distributed by Surovell *et al.* (*14*) among seven artificial elevational ASI bins. ASI bin elevation ranges for Cooper’s Ferry/Nipéhe are from Surovell *et al.* [(*14*), table S3]. Actual mapped elevations are shown in Fig. 2B.

ASI bin elevation range (masl)	ASI bin assignment by elevation
411.85–411.90	
411.80–411.85	
411.75–411.80	
411.70–411.75	
411.65–411.70	
411.60–411.65	
411.55–411.60	
411.50–411.55	**2**
411.45–411.50	**7**
411.40–411.45	**7**
411.35–411.40	**2**
411.30–411.35	**3**
411.25–411.30	**1**
411.20–411.25	**2**
411.15–411.20	
411.10–411.15	
411.05–411.10	
411.00–411.05	

#### Independent tests of stratigraphic integrity

For these reasons, the ASI score alone cannot demonstrate substantial vertical mixing at Cooper’s Ferry/Nipéhe. The score was calculated from 5-cm absolute-elevation bins that do not actually represent the site’s recorded stratigraphic relationships. Assessment of the mixing hypothesis must therefore rely on independent evidence from the site’s stratigraphy, chronology, pedogenesis, cultural features, and excavation record. If LU3 and LUB3 had been affected by vertical mixing at a scale sufficient to invalidate the site’s early chronology, then several expectations should follow. Radiocarbon ages should fail to preserve coherent older-with-depth structure; Bayesian modeling should identify substantial chronological disorder; cultural features should be blurred or homogenized; and pedogenic horizonation should be disrupted and inconsistent with intact soil development.

The Cooper’s Ferry/Nipéhe record does not meet these expectations. Radiocarbon ages from intact cultural features and other samples collected from in situ positions within the LU3 loess produce a coherent chronological structure when evaluated through Bayesian modeling (*17*, *18*). Although individual outlying ages occur, their presence does not by itself establish the nature or archaeological significance of stratigraphic mixing. Surovell *et al.* (*22*) and Surovell and Waguespack (*23*), for example, recognized substantially younger charcoal ages at Barger Gulch, including samples from the same stratigraphic contact and within centimeters of the inferred Folsom occupation surface yet interpreted these ages in light of independent archaeological and site-formation evidence and retained the Folsom age of the occupation. The broader chronostratigraphic pattern at Cooper’s Ferry/Nipéhe likewise remains ordered and internally coherent. If artifacts, charcoal, and faunal materials had been vertically displaced throughout LU3 and LUB3 on the scale implied by the ASI critique, then the modeled age structure should show substantial chronological disorder. Instead, the radiocarbon evidence supports the interpretation of successive temporally discrete cultural components preserved within a slowly accumulating loess deposit.

The preservation of distinct cultural features provides a second independent test of potential stratigraphic mixing. As Surovell *et al.* [(*14*), p. 12] note, “Of great importance is the presence of cultural features in sealed stratigraphic units associated with unambiguous occupation surfaces marked by sufficient artifact counts to allow their identification.” The Late Pleistocene LU3 and LUB3 deposits contain six pit features and one hearth formed on, or excavated into, loess surfaces during intervals of aeolian deposition. Continued loess accumulation subsequently buried these features, and their fills retain distinct boundaries, internal contents, and coherent stratigraphic relationships. They include concentrations of dozens to hundreds of artifacts, sets of stemmed points, faunal remains, oxidized deposits, charcoal, and contrasting sediments. Such features would not be expected to retain coherent boundaries, internal patterning, and stratigraphic relationships if LU3 and LUB3 had experienced postdepositional mixing at a scale sufficient to alter their vertical artifact distributions substantially. Even lesser feature-disruptive mixing should be expressed in blurred boundaries, disturbed internal sediment contrasts, displaced contents, or otherwise altered feature morphology, none of which is evident. Their preservation therefore indicates that postdepositional processes did not operate at a scale sufficient to disrupt feature morphology, destroy internal patterning, or redistribute feature contents beyond archaeological recognition.

The Rock Creek paleosol provides another independent line of evidence. LU3 and LUB3 preserve pedogenic horizonation, including a weak A horizon and Btk/calcic development expressed through carbonate filaments, coatings, hypocoatings, and pendants on and around artifacts and bone. These soil features record in situ pedogenic processes that developed after burial of earlier cultural materials. Substantial vertical disturbance should disrupt, blur, or erase this fragile soil architecture, particularly if the proposed mechanism required repeated displacement of archaeological materials through the deposit. Instead, the paleosol remains visible as a stratigraphic and pedogenic marker that can be correlated across the site and used to evaluate the relative position of cultural features and artifacts. Thickened carbonate coatings occur only on artifacts recovered from the lower portions of LU3 and LUB3, where they were overprinted by pedogenic Btk horizon development. This distribution is inconsistent with the ASI mixing interpretation, which requires artifacts to have moved both upward and downward through the deposit at a scale sufficient to blur the original artifact concentrations.

Artifact frequencies alone do not provide a reliable proxy for disturbance at Cooper’s Ferry/Nipéhe. In a setting characterized by repeated occupation during slow loess accumulation, changes in artifact frequency may result from differences in discard intensity, occupation duration, activity location, excavation sample size, feature density, or sediment accumulation rate. Demonstrating site disturbance requires identification of a plausible and verifiable postdepositional vector, specification of the processes by which materials moved, and evaluation of the larger expected consequences in the stratigraphic, chronological, pedogenic, and spatial records.

Rodent burrowing is a potential source of localized artifact displacement at Cooper’s Ferry/Nipéhe, but krotovina were readily identifiable in the early deposits because their fills contrast in color and texture with the surrounding lithostratigraphic matrix (*17*). In LU3 and LUB3, burrow traces commonly appear as darker, cylindrical, or branching fills that crosscut the lighter brown loess (Fig. 3). Excavators identified and delineated krotovina during excavation, removed and screened their fills separately from adjacent undisturbed sediment, and excluded artifacts recovered from burrows from the in situ artifact record (*17*, *18*).

**Fig. 3.**
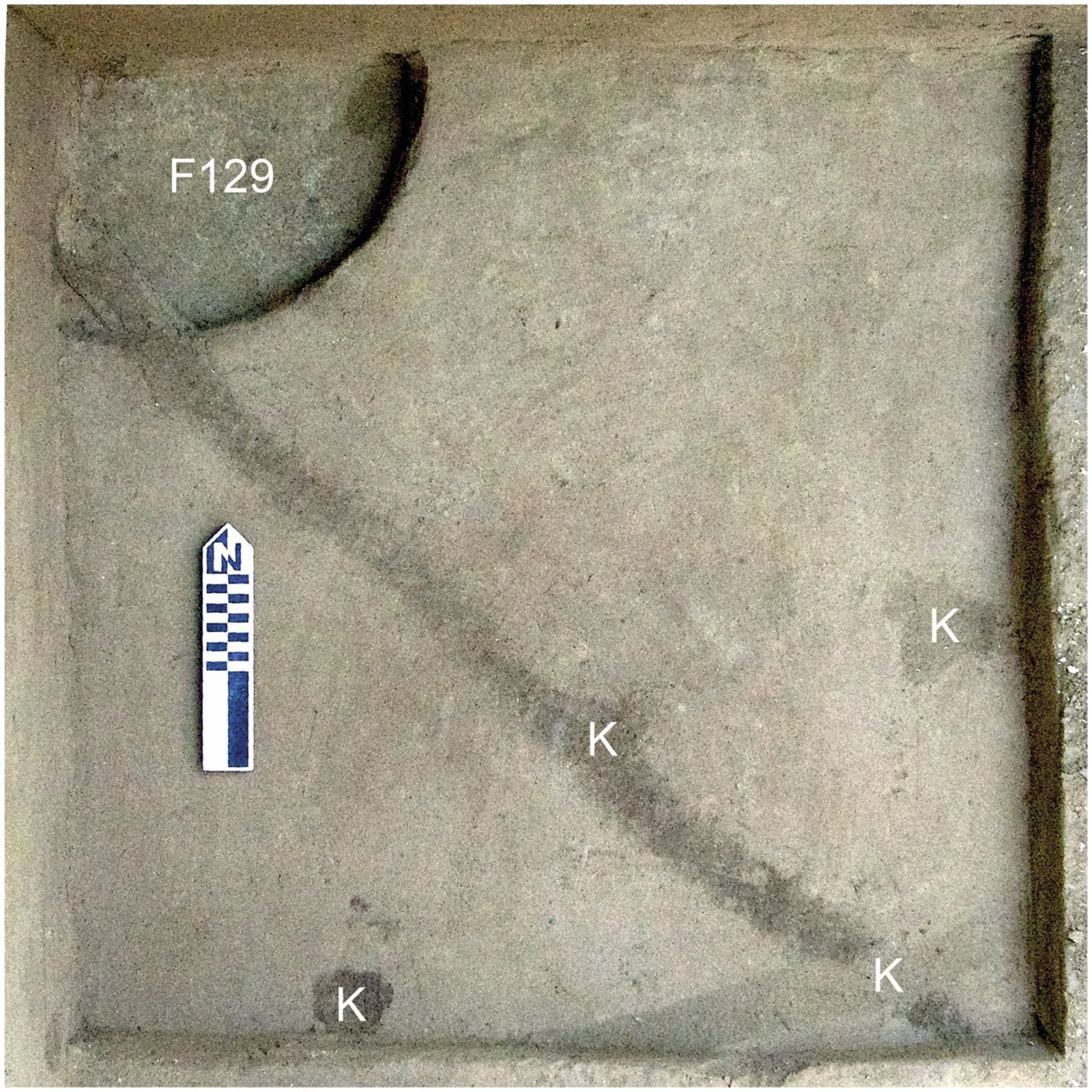
Krotovina visibility in LU3 loess. Multiple krotovina exposed in the LU3 loess of Area A near the bottom of F129 showing how distinctive the disturbed fill of the burrows is in coloring and texture from the surrounding undisturbed loess. Photo credit: Loren G. Davis, Oregon State University.

This approach is consistent with the earlier analysis of Surovell *et al.* (*22*) of vertical artifact dispersal at Barger Gulch, where they did not interpret vertical artifact distributions in isolation as evidence of disturbance. Instead, hypotheses concerning artifact movement and occupation history were evaluated against multiple independent lines of evidence, including artifact mass and inclination, diagnostic artifact distributions, refits and conjoins, local stratigraphic relationships, sedimentological evidence, and radiocarbon dating. Surovell *et al.* (*22*) used these observations collectively to reconstruct the nature and archaeological consequences of vertical dispersal at the site. Their analysis demonstrates the importance of establishing what site-formation processes actually produced a vertical artifact distribution before using that distribution to evaluate archaeological integrity. The methodological contrast is important: At Barger Gulch, the meaning of a vertical artifact distribution was established through independent archaeological and geological evidence rather than inferred from the shape of the artifact-frequency profile alone.

Claims of stratigraphic “mixing” at Cooper’s Ferry/Nipéhe are therefore misplaced for three reasons:

1) The ASI is a descriptive index of vertical artifact-frequency variation, not a direct measure of artifact movement or stratigraphic integrity; its interpretation depends on substantial and arbitrary assumptions about depositional, occupational, and site-formation processes.

2) Cooper’s Ferry/Nipéhe ASI analysis of Surovell *et al.* (*14*) treats 5-cm absolute-elevation bins as though they represent equivalent horizontal stratigraphic intervals across Area A, despite the irregular LU2 surface and the conformable accumulation of LU3 loess above it.

3) Independent chronological, stratigraphic, pedogenic, archaeological, and excavation evidence does not support the large-scale vertical mixing required by the interpretation of Surovell *et al.* (*14*).

### Coastal migration as a statistical model and the problem of inland dispersal

As we have demonstrated, the ASI analysis does not undermine the stratigraphic integrity or age of Cooper’s Ferry/Nipéhe, and the age of its ∼16,000–cal yr B.P. initial occupation thus has bearing on two earlier models developed by Surovell to evaluate the timing and geographic process of initial dispersal in the Americas. Surovell (*12*) designed a quantitative model of Pacific coastal migration to examine a specific problem in First Americans archaeology: Could people move southward along the Pacific coast and reach the latitude of Monte Verde II in southern Chile while leaving little comparable early archaeological evidence in inland North America? At the time, this was an important and reasonable question. Monte Verde II appeared to predate Clovis by ∼1200 to 1400 calendar years (*24*, *25*), whereas the North American record of sites older than Clovis remained limited, controversial, or difficult to evaluate. If people moved south of the Cordilleran ice sheet along the Pacific coast, then why did the earliest widely discussed archaeological evidence appear so far to the south rather than in western North America, closer to the likely point of entry?

Surovell (*12*) did not attempt to identify the actual route by which people entered the Americas or reached South America. Instead, he evaluated whether coastal migration alone could account for the apparent discrepancy between an early occupation in southern Chile and the limited inland archaeological record then known from North America. The model drew on human behavioral ecology to treat migration as a demographic and foraging process rather than as simple directional movement. Population growth, coastal movement, and inland dispersal were governed by changes in per-capita foraging returns and linked population growth to food-acquisition returns following the premise developed by Winterhalder *et al.* (*26*) that higher per-capita returns can support greater reproductive success. Coastal migration was therefore modeled as a process driven by population growth, population density, mobility, and the relative productivity of coastal and inland ecosystems.

The central question that Surovell (*12*) asked was whether a coastal population could reach the latitude of Monte Verde II, estimated at 13,400 km from the model’s entry point, before inland migration occurred in North America. The answer the model consistently predicted was that inland migration would begin first in the northern portion of the coastal expansion, and that no single adjustment to the model’s parameters could produce an arrival at Monte Verde before inland migration in North America. That is, if Pacific coastal migration had occurred, the earliest inland sites would be expected in western North America rather than in South America. More specifically, Surovell emphasized that “Where large rivers empty into the Pacific Ocean, perforating coastal mountain chains, inland ecosystems would have appeared very attractive to prehistoric populations, since continental waterways serve as linear ecotones where many terrestrial and aquatic resources are available” [(*12*), p. 588]. He identified the Columbia River, less than 200 km from the model’s entry point, as an “obvious corridor into continental North America” [(*12*), p. 588]. The model therefore provides a testable expectation: if people moved southward along the Pacific margin, early inland archaeological evidence should also occur in major river corridors that connected the coast to productive interior landscapes.

### Monte Verde II, Cooper’s Ferry/Nipéhe, and the absence argument

Monte Verde II has long occupied a central place in debates about the timing and process of the peopling of the Americas [e.g., (*27*)]. Its proposed age of ∼14,400 cal yr B.P. (*24*) placed people in southern Chile before the widespread appearance of Clovis in North America, casting doubt on a Clovis-First model of an inland initial occupation of the Americas after ∼14,000 cal yr B.P. However, the age of Monte Verde II and its location in far southern South America raised a persistent question about the apparent lack of comparably early archaeological evidence farther north along the expected route of dispersal. As part of these ongoing debates, Surovell’s model was designed to explore this question and to evaluate whether the early age of Monte Verde II could be inaccurate because no sites of comparable age were then known in the Columbia River basin. At the same time, the model predicted that earlier sites such as Cooper’s Ferry/Nipéhe should be found if the age of Monte Verde II was accurate. Cooper’s Ferry/Nipéhe therefore provides a direct means of evaluating the inland expectation generated by Surovell’s (*12*) model and whether Monte Verde II can be rejected out of hand on the basis of Surovell’s coastal-migration model alone.

Monte Verde II also provides a useful contrast to the ASI analysis. The MV-II occupation is a single cultural component sealed above and below by archaeologically sterile sediment (*24*, *25*). Its artifact distribution is therefore sharply bounded in the vertical dimension, with no evidence that objects moved into or out of the occupation layer after burial. Surovell *et al.* (*14*) did not apply the ASI to Monte Verde II, but an index calculated from stratigraphically meaningful artifact bins would be expected to approach the upper limit of the scale and to meet, or exceed, their criterion for “exceptional stratigraphic integrity.” This would not establish the integrity of Monte Verde II by itself. That conclusion rests on the site’s stratigraphy, sedimentology, archaeological associations, and chronology. But it illustrates the larger problem: The ASI merely describes a vertical artifact pattern, whereas the actual evidence for integrity comes from the independently documented site record.

The Cooper’s Ferry/Nipéhe site lies within the Columbia–Snake–Salmon River system (Fig. 4), a major route from the Pacific coast into continental North America, and its intact stratigraphic record documents human occupation there by at least ∼16,000 cal yr B.P. Its age predates the proposed ∼14,400 cal yr B.P. occupation at Monte Verde II by ∼1600 years. Its location and age therefore provide the kind of early inland evidence that Surovell’s (*12*) model indicated should exist if coastal populations also dispersed through accessible river corridors. To be clear, this correlation does not establish the age, preservation, or interpretation of Monte Verde II, nor does it demonstrate a direct historical connection between Cooper’s Ferry/Nipéhe and southern Chile.

**Fig. 4.**
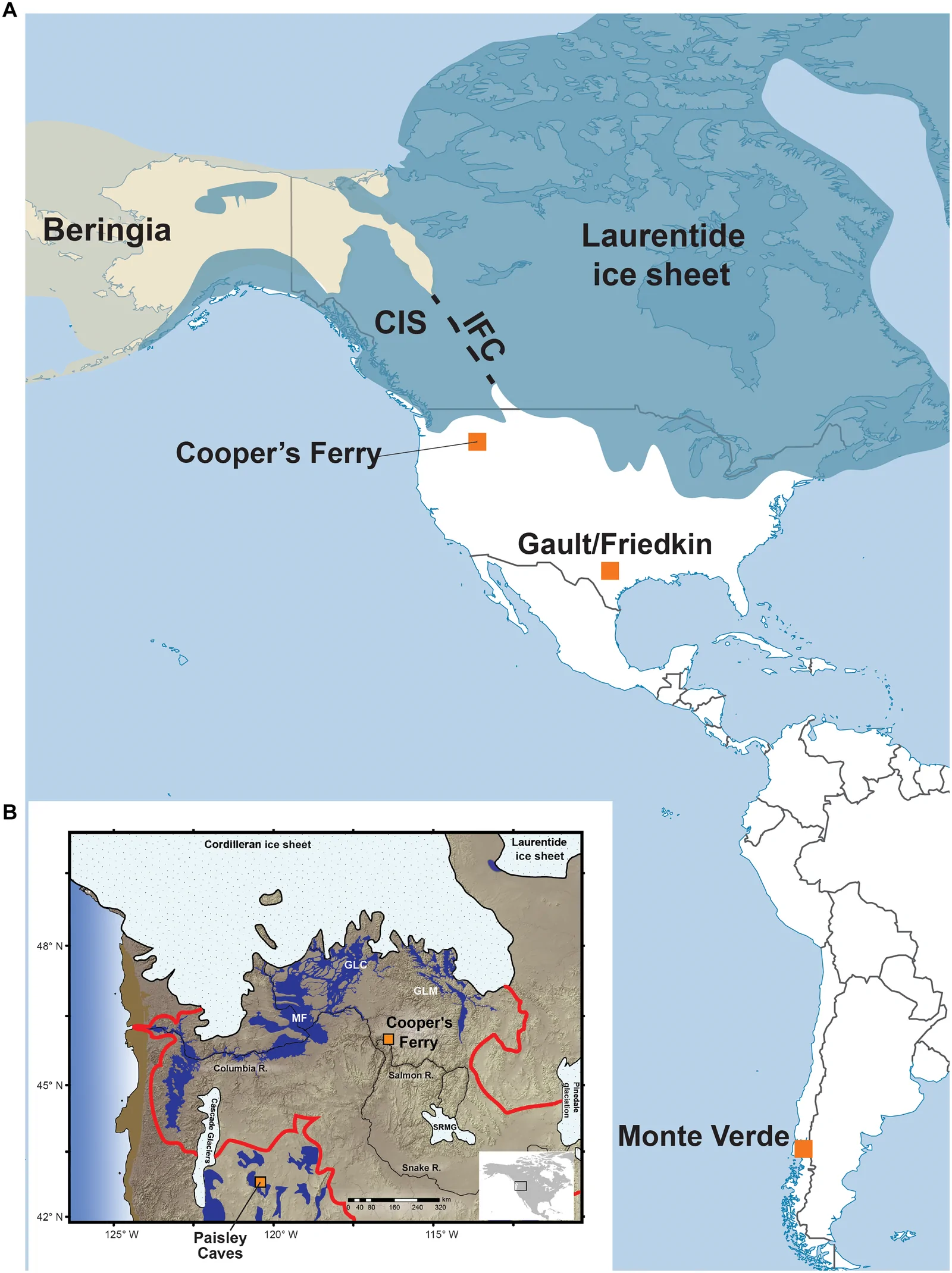
Continental and regional context of Cooper’s Ferry/Nipéhe. (**A**) Map of North and South America showing the locations of Cooper’s Ferry/Nipéhe, Gault/Friedkin, and Monte Verde. (**B**) Locations of Cooper’s Ferry/Nipéhe and Paisley Caves in and adjacent to the Columbia River drainage basin (extents outlined in red).

Monte Verde II must continue to be directly evaluated from its own stratigraphy, archaeological associations, site-formation history, and chronometric evidence (*24*, *25*, *27*–*30*). However, Together, the inland expectation generated by Surovell’s (*12*) model and the Cooper’s Ferry/Nipéhe archaeological and geoarchaeological record change the broader context in which Monte Verde II is evaluated. Monte Verde II no longer appears anomalously early simply because comparably early inland evidence in North America was once unknown. Cooper’s Ferry/Nipéhe documents human occupation by at least ∼16,000 cal yr B.P. in the Columbia–Snake–Salmon River system, near a major route from the Pacific coast into the continental interior. The former absence of such a record can no longer be used to evaluate the reliability of Monte Verde II; its interpretation must rest on the evidence at Monte Verde II itself.

### Cooper’s Ferry/Nipéhe and inland population expansion in North America

The implications of Surovell’s (*12*) earlier modeling extend beyond the geographic location of early inland occupation. If Cooper’s Ferry/Nipéhe provides the kind of early inland evidence anticipated by this model, then its ∼16,000 cal yr B.P. age also raises a related question about the pace at which those populations could have expanded across North America. Surovell’s (*15*) demographic model provides a useful framework for evaluating that question. Surovell (*15*) also modeled the rate at which initial human populations could have—and, under the model’s assumptions, probably would have—expanded across North America in less than a thousand years. Although the model was explicitly designed to explain the rapid expansion of populations using Clovis technology after entering North America through an ice-free corridor, its underlying assumptions about mobility, fertility, and population growth may also be relevant to an earlier initial occupation entering through the Columbia River basin.

The essential predictions of the model are that foragers entering an unpopulated landscape should maximize both high residential mobility and high reproductive rates. For CPT populations to expand across North America in the ∼700 years Surovell suggests that it took to occupy the continent would require annual population increases of ∼1 to 3% per year [see discussion in (*15*), p. 504; compare with (*31*)]. This is an extremely high rate, but one required by the relatively short timeframe. Slightly smaller rates of population growth would produce slower but still relatively rapid rates of population expansion. Paleogenetic estimates of the time it took for this initial population to spread across the Americas are on the order of 2000 to 3000 years (*32*–*34*). Even these somewhat longer estimated time spans require relatively high annual rates of population growth, but rates that remain within the parameters modeled by Surovell (*15*).

So, what does the pairing of these estimated expansion rates, the early inland expectation generated by Surovell (*12*), and an age of ∼16,000 cal yr B.P. for the initial occupation of Cooper’s Ferry/Nipéhe tell us about inland expansion in North America? First, they suggest that other Late Pleistocene sites should be found in or near the Columbia River drainage. Not unexpectedly, Paisley Caves, 1 of 10 AUP sites in North America identified by Madsen *et al.* (*21*), lies just outside the upper Columbia River tributaries in southeastern Oregon (Fig. 4B) (*35*–*37*). Dated human fecal remains at the site suggest that occupation began between ∼14,500 and ∼14,000 cal yr B.P., or only about 1500 years after the initial occupations of Cooper’s Ferry/Nipéhe (*17*, *18*).

Second, these models suggest that pre-Clovis sites should be widely distributed across the unglaciated portions of North America before the opening of an interior ice-free corridor. Consistent with this expectation, the other nine AUP sites considered by Madsen *et al.* (*21*) are distributed from Florida to Pennsylvania and Virginia and from Wisconsin to Texas and New Mexico. Their geographic distribution is consistent with rapid expansion across the unglaciated continent following one or more Pacific coastal entry routes. Whether there was only a single entry point near the mouth of the Columbia River remains unknown, and other entry points along the coast are also likely. Regardless, an early entry through the Columbia River basin followed by rapid expansion across North America is consistent with expectations generated by the models of Surovell (*12*, *15*).

Several computer models and statistical analyses have been used to evaluate questions about the timing, geographic process, and rate of the initial peopling of the Americas (*12*, *14*, *15*). Their usefulness depends on whether their assumptions and predictions can be evaluated against the archaeological evidence to which they are applied.

The ASI developed by Surovell *et al.* (*14*) is a numerical description of vertical artifact-frequency variation, not a direct measure of artifact movement, postdepositional disturbance, or stratigraphic integrity. Differences in ASI values can only be interpreted as differences in mixing when the compared sites have broadly comparable depositional histories, artifact assemblages, occupation histories, excavation strategies, and site-formation processes. Those conditions are not met among the sites included in the analysis of Surovell *et al.* (*14*). The ASI can be useful for describing the degree of vertical discontinuity in a binned artifact-frequency profile, but it cannot by itself identify the processes that produced that pattern or measure the amount of artifact movement that occurred.

Even on the interpretive terms of Surovell *et al.* (*14*), none of the sites that they evaluated approaches complete vertical discontinuity. The ASI results therefore do not provide a straightforward basis for distinguishing “exceptional” from intermediate stratigraphic integrity without independent site-specific evidence. Such evidence is required to determine whether a smooth or continuous artifact-frequency profile resulted from postdepositional mixing, repeated occupation during sediment accumulation, changing discard intensity, shifting activity areas, variation in sedimentation rates, excavation-bin construction, or other processes.

At Cooper’s Ferry/Nipéhe, the ASI analysis does not provide a valid basis for inferring large-scale vertical mixing. The artificial object distribution used by Surovell *et al.* (*14*) to assign an intermediate ASI value to the site was constructed from 5-cm absolute-elevation bins that do not correspond to the artifacts’ actual stratigraphic positions. The resulting ASI calculation therefore evaluates a vertical distribution created by the binning procedure rather than the site’s recorded artifact distribution. Independent stratigraphic, pedogenic, archaeological, chronological, and excavation evidence likewise does not support the scale of mixing required by their ASI interpretation. The ∼16,000–cal yr B.P. occupation at Cooper’s Ferry/Nipéhe cannot reasonably be dismissed as the product of large-scale artifact mixing.

These results also bear on two earlier models developed by Surovell. The coastal-migration model of Surovell (*12*) predicted that, if people moved southward along the Pacific margin, inland dispersal should begin in western North America before coastal populations reached the latitude of Monte Verde II. It specifically identified the Columbia River as an accessible route into continental North America. Cooper’s Ferry/Nipéhe now provides the type of early inland evidence that the model indicated should exist within such a river corridor. This does not establish the age, preservation, or interpretation of Monte Verde II, nor does it demonstrate a direct historical relationship between Cooper’s Ferry/Nipéhe and southern Chile. It does mean, however, that the former absence of early inland archaeological evidence in the Columbia River basin can no longer be used to evaluate the reliability of Monte Verde II. Its interpretation must rest on the archaeological, geological, and chronometric evidence from Monte Verde II itself.

Similarly, while Surovell’s (*15*) model of rapid population expansion was designed to explain the spread of Clovis populations entering North America through an ice-free corridor, its underlying demographic assumptions may also be relevant to earlier populations entering through Pacific coastal routes. The relatively narrow age range and broad geographic distribution of AUP sites across the southern latitudes of North America (*21*), together with paleogenetic evidence for rapid population expansion beginning ∼17,000 to 15,000 cal yr B.P. (*32*–*34*, *38*–*40*), are consistent with rapid Late Pleistocene expansion across the continent.

At Cooper’s Ferry/Nipéhe, the stratigraphic, archaeological, and chronological evidence indicates that the ∼16,000–cal yr B.P. occupation cannot reasonably be dismissed as the product of large-scale artifact mixing. When a model ignores the multidisciplinary data it claims to explain, its formal structure is beside the point. It becomes magical thinking presented as scientific analysis. Models are hypotheses to be tested against site data, not authorities that can override the archaeological record.

### Provenance statement

The archaeological materials and data discussed in this manuscript were recovered from Cooper’s Ferry/Nipéhe (10IH73), located on an alluvial terrace at the confluence of Rock Creek and the lower Salmon River in western Idaho, within the traditional homeland of the Nimiipuu. The materials considered here were collected during archaeological excavations conducted in 1997 and from 2009 to 2018 in Areas A and B under the direction of L.G.D., in cooperation with the Bureau of Land Management and the Nez Perce Tribe. Fieldwork was carried out by Oregon State University archaeology field school staff, students, and interns, with participation by members of the Nez Perce Tribe. These excavations recovered cultural materials, faunal remains, charcoal, cultural pit features, hearth deposits, and associated sediment samples from stratified Late Pleistocene and early Holocene deposits. The archaeological context and authenticity of these materials were evaluated through controlled excavation, in situ mapping, stratigraphic recording, geoarchaeological analysis, artifact and feature analysis, archaeofaunal analysis, radiocarbon dating, luminescence dating, and Bayesian chronological modeling, as reported in (*16*–*20*). Chronological evaluation was based on accelerator mass spectrometry radiocarbon dating of charcoal and bone, optically stimulated luminescence dating of sediment samples, and Bayesian chronological models that incorporated stratigraphic information and outlier analysis. This manuscript reports and analyzes no human remains. The Cooper’s Ferry/Nipéhe archaeological collection is curated at the Oregon State University Department of Anthropology. Published artifact, stratigraphic, chronological, and analytical data used in this manuscript are available in the cited publications and their supplementary materials. Access to the curated physical collections may be requested through the Oregon State University Department of Anthropology, subject to applicable curation policies, research permissions, and consultation requirements.
